# Clinicopathological Features, Staging Classification, and Clinical Outcomes of Esophageal Melanoma: Evaluation of a Pooled Case Series

**DOI:** 10.3389/fonc.2022.858145

**Published:** 2022-07-01

**Authors:** Haiyan Sun, Ningning Zhu, Lei Gong, Lan Lan, Zhentao Yu, Zhanyu Pan

**Affiliations:** ^1^ Department of Integrative Oncology, Tianjin Medical University Cancer Institute and Hospital, National Clinical Research Center for Cancer, Key Laboratory of Cancer Prevention and Therapy of Tianjin City, Tianjin, China; ^2^ Department of Esophageal Cancer, Tianjin Medical University Cancer Institute and Hospital, National Clinical Research Center for Cancer, Key Laboratory of Cancer Prevention and Therapy of Tianjin City, Tianjin, China

**Keywords:** esophagus, melanoma, classification, survival analysis, predictor

## Abstract

Studies that have attempted to validate the staging systems and the predictors of survival for patients with primary malignant melanoma of the esophagus (PMME) have been underpowered given their scarcity and small scale. We aimed to review a large number of PMME cases to know more about its clinicopathological features, TNM staging systems, and survival predictors of PMME. Case reports on PMME were extracted from PubMed/Medline through bibliography search and our center. A total of 287 PMME cases were identified. The majority of the patient population was male (72.08%). The most common location of PMME was the lower esophagus (50.62%) and middle esophagus (35.39%). Among the patients, 82.28% received surgical intervention. The median overall survival (OS) duration was 15 months (0.5–244). The American Joint Commission on Cancer staging classification (AJCC) for the mucosal melanoma of the upper aerodigestive tract with stage IVB and IVC integrated in stage IVA showed better distribution of OS than that for esophageal carcinoma. T stage, N stage, and surgery had significant impacts on OS duration in univariate analysis. However, only T stage and N stage were identified as independent factors for OS duration in the multivariate Cox models. PMME is an aggressive tumor with poor prognosis. The AJCC staging system for mucosal melanoma with stage IVB and IVC integrated in stage IVA may be a better option for staging PMME patients. T stage and N stage are independent factors for OS.

## Introduction

Cutaneous melanoma accounts for 95% of all reported malignant melanoma cases (1). Extracutaneous melanoma is more aggressive than cutaneous melanoma with its poor prognosis (2, 3). Primary malignant melanoma of the esophagus (PMME) is a rare disease accounting for approximately 0.5% of all extracutaneous melanomas (4–7). Bisceglia (8) reported that only 337 cases of PMME have been published from 1906 to the end of October 2010. There are no standardized staging systems and treatment guidelines for PMME available now. The median survival duration of PMME patients ranges from 8 to 34.5 months (8–11). Several questions about PMME remain unanswered, including its clinicopathological characteristics, staging, and prognosis.

The rarity of PMME have underpowered the attempt to validate the staging systems and to identify predictors of survival for PMME, which in turn further hindered the prospective randomized trials. For the benefit of PMME patient, we aimed to review a large number of PMME cases to know more about its clinicopathological features, TNM staging systems, and survival predictors of PMME. There is no recognized staging system for PMME. In this article, two TNM staging systems were mentioned. TNMe stage represented the staging for esophageal carcinoma according to the 8^th^ edition of the American Joint Commission on Cancer staging classification (AJCC) for esophageal carcinoma (11), while TNMm stage referred to mucosal melanoma staging according to 8^th^ edition of the AJCC Cancer staging for mucosal melanoma of the upper aerodigestive tract (12).

In the mucosal melanoma classification, the T classification of T1 and T2 and TNM classification of Stages I and II are omitted. Stage T3 is defined as tumor limited within the submucosal layer, and T4a is the tumor invading deep soft tissue, cartilage, bone, or overlying skin. T4b is defined as tumor invading brain, dura, skull base, lower cranial nerve, masticator space, carotid artery, prevertebral space, mediastinal structure, cartilage, and skeletal muscle or bone. The details of mucosal melanoma classification and esophageal carcinoma are elaborated in **Supplementary Table 1**.

## Materials and Methods

### Patient Selection

The PMME cases reviewed in this study included patients retrieved from the literatur. By conducting a PubMed/Medline bibliography search of the terms “melanoma” and “esophagus”, we retrieved 293 cases (**Supplementary Table 2**) of PMME reported from January 2007 to December 2021. After removing 6 cases (9) overlapped with the 17 cases from the same cancer center reported by Gao et al (12), a total of 287 PMME cases were identified in the study. The study was conducted in accordance with the guidelines approved by the Ethics Committee of the Tianjin Medical University Cancer Hospital and Institute.

**Figure 1:**
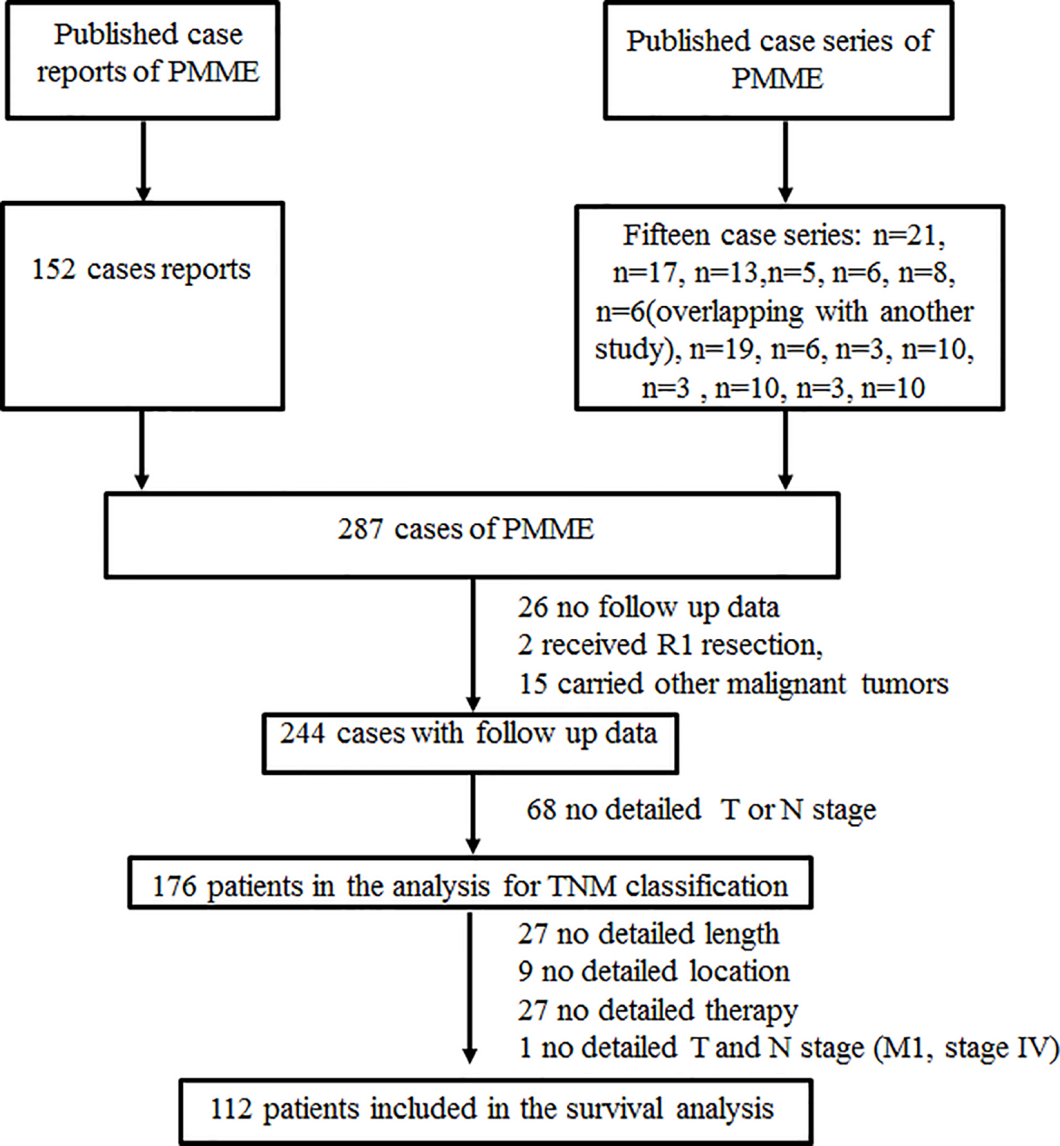
Schematic of the selection of the esophageal melanoma patients.

The following clinicopathological data were retrieved from medical records in our center and extracted from published reports and studies: age, sex, race, tumor location, tumor number, tumor size, symptoms, histological differentiation, lymphovascular invasion, perineural invasion, soft tissue invasion, depth of the invasion of the primary tumor, N stage, metastasis, TNMe stage, TNMm stage, immunohistochemical features, mutational status, surgery, chemotherapy, radiotherapy, immunotherapy intervention, accompanied tumor, and survival data. Race/ethnicity was based on the countries of origin of the authors if unspecified. The details of mucosal melanoma classification are elaborated in **Table 1**. The exclusion criteria for survival analysis were as follows: did not receive R0 resection, carried other malignant tumors, had no complete data or follow-up data. Moreover, patients were only included in the analysis of the staging system when they had complete data, including T stage, nodal status, distant metastases, and follow-up data, allowing us to restage according to the 8th AJCC classifications for the esophageal and mucosal melanoma of the upper aerodigestive tract. Survival analysis was conducted based on gender, tumor location, tumor number, tumor size, TNM stage, surgery, chemotherapy, radiotherapy, and immunotherapy intervention. Consequently, 176 patients were included in the study in accordance with TNM classification, with only 112 of them included in the survival analysis. Selection of patients with esophageal melanoma was shown in **Figure 1**.

**Table 1:** American Joint Comission on Cancer staging for mucosal melanoma (upper aerodigestive) 8th edit.

STAGE GROUPING			
Stage III	T3	N0	M0
Stage IVA	T4a	N0	M0
	T3, T4a	N1	M0
Stage IVB	T4b	Any N	M0
Stage IVC	Any T	Any N	M1

### Statistical Analysis

Statistical analyses were performed using SPSS 26.0 (SPSS Inc., Chicago, IL, USA). Numerical variables were expressed as the mean ± SD. TNM staging on survival analysis for the outcome measure was based on Kaplan–Meier methods. Univariate multivariate Cox proportional hazards regression analysis was performed to evaluate the two staging systems and other prognostic factors, including age, gender, tumor location, length, T stage, N stage, metastasis, and therapy intervention. *P*<0.05 was considered to indicate a statistically significant difference.

## Results

### Symptoms and Clinical Characteristics

The clinicopathological characteristics of the patients are listed in **Table 2**. The median age was 63 (36–91) years and the majority of the patient population was male (204/283, (72.08%)) in term of gender and Asian (201/283, 71.73%) geographically. Twenty-nine patients were misdiagnosed, including 22 patients with esophageal squamous cell carcinoma, 6 with esophageal adenocarcinoma, and one with plasma cell myeloma. Histopathological examination revealed the presence of *in situ* melanoma in 11 cases. Fifty-four (18.82%) cases presented with multifocality or satellite lesions, with median tumor length of 5.25 cm (0.5 to 17).

**Table 2:** Clinicalpathological characteristics of 287 cases of esophageal melanoma.

Characteristics	Parameters
Age (n=277)	
<60	100 (36.10%)
≥60	177 (63.90%)
Gender (n=283)	
Female	79 (27.92%)
Male	204 (72.08%)
Geography (n=283)	
Asian countries	203 (71.73%)
Caucasian countries	79 (27.92%)
Others (Brazil)	1 (0.35%)
Tabacco use (n=65)	
Yes	34 (52.31%)
No	31 (47.69%)
Alcohol use (n=61)	
Yes	25 (40.98%)
No	36 (59.02%)
Position (n=243)	
Upper esophagus	9 (3.70%)
Upper-middle	3 (1.23%)
Middle esophagus	86 (35.39%)
Middle-lower esophagus	19 (7.82%)
Lower esophagus	123 (50.62%)
Whole esophagus	3 (1.23%)
Gross (n=194)	
Polypoid/solid/exophytic/nodules/lump forming/bulky/localized/pedunculated tumor/protrusions	148 (76.29%)
Slightly elevated/flat mass	30 (15.46%)
Ulcer	11 (5.67%)
Fungating	5 (2.57%)
Tumor number (n=287)	
One	233 (81.18%)
Multifocality/satellite lesions	54 (18.82%)
Symptoms (n=177)	
Dysphagia	167 (94.35%)
Weight loss	20 (11.30%)
None	31 (17.54%)
Others (retrosternal pain, epigastralgia, chest pain, dyspepsia, asthenia and anorexia)	31 (17.54%)
Symptom duration average (month) (n=167)	3.38
Immunohistochemical staining (n=137)	
Melan-A	66
HMB45	115
S-100	124
Ki-67 (n=19)	
Low	6 (31.58%)
High	15 (68.42%)
Gene mutational status	
* KIT* (n=9)	3 (33.33%)
* RAS* (n=10)	7 (70%)
* BRAF* (n=13)	4 (30.77%)
Tumor length average (n=210) (cm)	6.19
Metastasis (n=213)	
Yes	20 (9.38%)
No	193 (90.62%)
Lymphatic vessel and perineural invasion (n=39)	
Lymphatic	21 (53.85%)
Vessel	19 (48.72%)
Perineural	1 (2.56%)
Surgery (n=254)	
Yes	209 (82.28%)
No	45 (17.72%)
Surgery complication (n=209)	29 (13.88%)
Chemotherapy (n=180)	
Yes	79 (43.89%)
No	101 (56.11%)
Radiotherapy (n=180)	
Yes	33 (18.33%)
No	157 (81.67%)
Immunotherapy (n=180)	
Yes	43 (23.89%)
No	137 (76.11%)
Accompanied malignant tumors (n=287)	16 (5.57%)

### Treatment and Survival

A total of 209 (82.28%) patients received surgical intervention. Among these cases, three underwent endoscopic submucosal dissection and three received R1 resection. Surgical complication occurred in 29 (13.88%) patients, and three died of surgery. The median number of dissected lymph nodes was 20 (1-105). 79 patients (43.89%) received chemotherapy, 33 patients (18.33%) went through radiotherapy, and 2 underwent thermoradiotherapy. Immunotherapy was recorded for 43 patients (23.89%).

**Figure 2:**
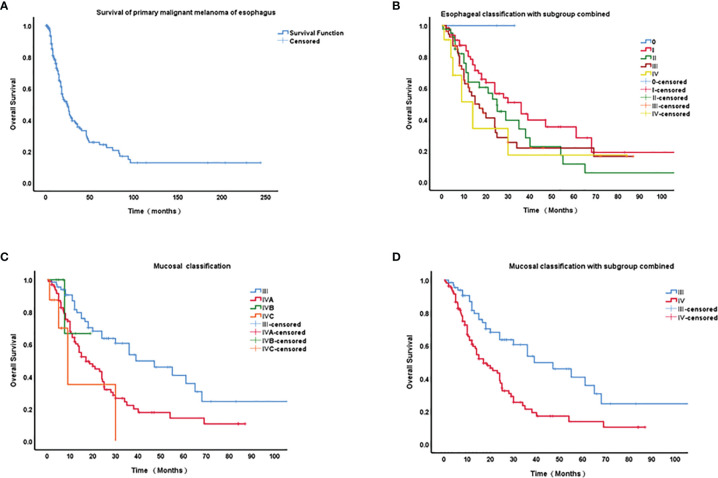
Kaplan–Meier curves of survival of primary malignant melanoma of esophagus (PMME) and different staging classifications for PMME patients. **(A)** survival of 182 cases with PMME; **(B)** The 8th AJCC classifications of esophageal carcinoma for PMMEs. **(C)** The 8th AJCC classifications of mucosal melanoma. **(D)** Mucosal classification with IVB and IVC integrated into IVA.

Survival data are shown in **Figure 2A**. The median overall survival (OS) duration was 12.9 months (0.5-244 months, n = 226). The 1-, 2-, 3-, 4- and 5-year survival rates were 55.75%, 30.97%, 15.93%, 9.73%, and 5.31%, respectively.

### Correlation of Different Classification Systems With Survival

A final cohort of 176 patients with a known stage was included in the analysis of TNM classification for PMME. The Kaplan–Meier curves for OS based on AJCC staging systems for esophageal cancer and mucosal melanoma are plotted in **Figures 2B–D**. Univariate prognostic factor analysis by Cox proportional-hazards regression for OS was performed to identify the staging classifications of tumors on the basis of outcome (**Table 3**). Significant differences have been observed among the TNMe stages 0(n=2), I(n=66), II(n=41), III(n=56), and IV(n=11) (**Figure 2B**) as well as TNMm stages III(n=67), IVA(n=96), IVB(n=6) and IVC(n=8). Patients with TNMe stage II have a poorer survival compared with those patients having stage I (0.488 and 0.704, respectively in **Table 3**). However, there was overlap and intersection between the curves of stages II, III, and IV disease. The hazard ratio (HR) of stage II was comparable to that of stage III (0.704 and 0.816, respectively, shown in **Table 3**). A better distribution of OS for PMME with *p*<0.001 was observed when the staging system for mucosal melanoma of the upper aerodigestive tract was employed (**Figure 2D**). The HRs of stage IVA, IVB and IVC were 2.472, 7.122, 10.056 compared with stage III (**Table 3**), which meant patients with stage IVC had a greater relative risk for death than those with stage IVB and those with stage IVB had a greater relative risk for death than those with stage IVA. Notably, stage IVA and other stages were overlapped. When stage IVB and IVC were combined with stage IVA because of its rarity, the overlap disappeared and survival curves became well distinguishable between stages (**Figure 2C**). HRs were also observed with significant difference.

**Table 3:** Univariate Prognostic Factor Analysis for overall survival by Cox Proportional-hazards Regression.

	TNM stage	*p* value	HR (95%CI)
Esophageal classification between stages 0^#^, I, II, III, and IV	0	0.060	0.000
	I		0.488 (0.203,1.170)
	II		0.704 (0.287,1.726)
	III		0.816 (0.341,1.953)
	IV		Reference
Mucosal classification between III, IVA, IVB, and IVC	III	<0.001	Reference
	IVA		2.472 (1.469, 4.160)
	IVB		7.122 (0.915,55.429)
	IVC		10.056 (2.823,35.822)

^#^stages 0: high grade dysplasia.

### Predictive Factors for OS

A total of 112 patients were included in the survival analysis, shown in **Table 4**, T stage, N stage, and surgery had significant impacts on OS time in univariate analysis. The predictive variables of metastasis and surgery were no longer statistically significant and only T stage and N stage were independent factors for OS in the multivariate Cox models.

**Table 4:** Univariate and Multivariate Analyses of Predictive Factors for Overall Survival.

	Univariate analysis		Multivariate analysis	
	p	HR	p	HR
Age	0.997	1.000 (0.973,1.028)	0.157	1.025 (0.991,1.060)
Gender	0.462	0.816 (0.474,1.404)	0.167	0.649 (0.351,1.199)
Num	0.877	0.952 (0.513,1.768)	0.450	1.056 (0.545,2.046)
Position	0.662		0.871	
Tumor length	0.624	0.879 (0.524,1.474)	0.358	0.753 (0.411,1.379)
T	0.070	1.233 (0.982,1.547)	0.047	1.385 (1.005,1.908)
N	0.030	1.777 (1.052,3.002)	0.019	2.189 (1.140,4.205)
M	0.074	5.262 (0.686,40.383)	0.329	5.013 (0.197,127.799)
Surgery	0.018	0.203 (0.046,0.884)	0.559	0.509 (0.053,4.896)
Chemotherapy	0.388	0.788 (0.458,1.356)	0.191	0.645 (0.334,1.245)
Radiotherapy	0.113	0.336 (0.082,1.379)	0.131	0.295 (0.060,1.439)
Immunotherapy	0.618	0.860 (0.476,1.555)	0.553	0.815 (0.415,1.602)

## Discussion

Given the extremely rare incidence of PMME, studies that involve high numbers of PMME cases are scarce. In this study, we used a large cohort to evaluate the validity of different staging classifications in predicting OS and to determine the predictive factors for the survival of PMME patients. We found that the mucosal staging system with stage IVB omitted and integrated into stage IVA is a valid staging system for PMME. T stage and N stage are two independent prognostic factors for OS.

PMME is an aggressive neoplasm with a five-year survival rate of only approximately 4% (9, 13). In this review, we found that the median survival duration is 15 months and that the 1-, 2-, and 5-year survival rates are 55.75%, 30.97% and 5.31%, respectively. Bishop (14) reported that the five-year survival rates of patients with mucosal melanoma are significantly lower than those with cutaneous melanoma. Gao (12) reported a median survival of 18.1 months and 1- and 5-year survival rates of 51% and 10%, respectively. Consistent with our present findings, Chalkiadaki (15) reported a mean survival duration of 13 months for 110 PMME patients. Although our findings showed that the surgical resection rate among patients with PMME reached 82.28% (209/254), the majority of the patients died after surgery from disseminated disease.

Currently, there is no standardized staging system for PMME available. Mucosal melanomas arise directly from resident melanocytes in the mucosa. Due to the biological aggressiveness of mucosal melanomas, the relatively advanced nature at diagnosis, and the molecular features different from those classically associated with cutaneous melanoma, the staging is generally not based on the cutaneous malignant melanoma staging. Mucosal melanoma is introduced in the 8^th^ edition of AJCC cancer staging manual, for separate consideration from other mucosal-based lesions. The system omits TI and T2 categories, and even small superficial lesions have an overall poor prognosis. Stage IVB represents extensive local invasion for which treatment often is a nonsurgical approach for local palliation. Stage IVC denotes distant metastatic disease. Only a few authors (11, 14, 16) used mucosal melanoma staging classifications for PMME. In contrary, most reports utilized the staging system for esophageal carcinoma. The coexistence of these two parallel systems in practice has resulted in confusion for clinicians. A previous study conducted at our center suggested that the staging system for the mucosal melanoma of the upper aerodigestive tract might be a better option for staging patients with PMME than that for esophageal classification (16). In this study, we found that the difference of PMME patients between TNMe stages were not significant. The curves of stages II, III, and IV disease were overlapped. There was dramatic overlap among Stage IVB and IVBC disease and other stages in the mucosal staging system. These results revealed the shortcomings of the current staging classification. Therefore, changes of the current staging classification are urgently required. Given the rarity of stage IVB and IVC disease, we integrated stage IVB and IVC into stage IVA, resulting in disappearance of overlap and a worse survival from stages III to IV. These results together suggested that the staging classification for the mucosal melanoma of the upper aerodigestive tract with stage IV combination might be a better solution for staging patients with PMME.

Age, stage, the presence of lymph nodes metastasis and surgery are considered as main possible independent predictors. Cheung et al. (17) reviewed reports on primary gastrointestinal melanomas retrieved from the Surveillance, Epidemiology and End Reports database between 1973 and 2004. They found that increasing age, stage, and the presence of lymph nodes are independent predictors of lower OS, whereas surgical resection is an indicator of a significantly better outcome. The present study only revealed the T and N stages are independent factors for the OS of PMME patients. Although surgery was associated with a reduced risk of death in univariate analysis, multivariate analysis did not show survival benefits. This result may be related to the selection bias associated with the online database and the selective reports on early-stage disease (only two cases of metastasis were reported with complete data). Other authors (14, 18) reported that lymph node metastasis is an independent predictive factor for the postoperative survival of patients with PMME. Gao (12) and Harada (11) also found that patients with lymph node metastasis have poorer survival than those without metastasis even though there was no significant difference. In addition, Weiner (19) retrieved 56 case reports of PMME published between 2004 and 2011 from the National Cancer Database and showed that metastatic disease and regional disease are associated with worse survival.

The present study has several limitations despite its large cohort. First, the study is a retrospective analysis and all data were collected from PubMed/Medline bibliography. We did not list the cases included in the study of cancer registries that covered several decades, such as the cases reviewed by Weiner (19) Sekine (20) and Cote (21), which may overlap with the cases included the present study. Second, patients with missing data were excluded from the analysis, leading to selection bias. Third, given the fact that their IVB and IVC groups were so small, the conclusion that integrating IVA, IVB and IVC into a single mucosal melanoma staging IV may be overstated. Finally, we could not analyze patient comorbidities, performance status, histological differentiation, lymphovascular invasion, neural invasion, and soft tissue invasion or other potential prognostic factors for survival.

## Conclusion

PMME is an extremely aggressive tumor with poor prognosis. The staging system for the mucosal melanoma of the upper aerodigestive tract with stage IVB and IVC integrated into stage IVA may be a better option for staging patients with PMME than current esophageal classification. T and N stages are independent factors for OS.

## Data Availability Statement

The original contributions presented in the study are included in the article/**Supplementary Material**. Further inquiries can be directed to the corresponding authors.

## Author Contributions

HS, NZ: design and initiation of the study, quality control of data, data analysis and interpretation, and manuscript preparation and editing. LG: data acquisition. LL: conduct the editing and critical revision. ZY: Participate in the writing of manuscripts or critically revise important intellectual content; ZP: Significant contributions to data analysis and interpretation. All authors contributed to the article and approved the submitted version.

## Funding

This study was supported by the Tianjin Health and Family Planning Commission program (No.2017166).

## Conflict of Interest

The authors declare that the research was conducted in the absence of any commercial or financial relationships that could be construed as a potential conflict of interest.

## Publisher’s Note

All claims expressed in this article are solely those of the authors and do not necessarily represent those of their affiliated organizations, or those of the publisher, the editors and the reviewers. Any product that may be evaluated in this article, or claim that may be made by its manufacturer, is not guaranteed or endorsed by the publisher.
