# Supplementary material for: Clinicopathological Features, Staging Classification, and Clinical Outcomes of Esophageal Melanoma: Evaluation of a Pooled Case Series
Source: Front Oncol. 2022 Jul 1;12:858145. doi: 10.3389/fonc.2022.858145 (PMC9283823; doi:10.3389/fonc.2022.858145)
Supplement: Supplementary file 1 [file Table_1.docx]

Supplementary Table 1 Tumor-node-metastasis classification by the AJCC staging for melanoma of the upper aerodigestive tract and esophageal cancer

|  |  | AJCC staging for melanoma of the upper aerodigestive tract | AJCC staging for esophageal cancer |
| --- | --- | --- | --- |
| T | Tx | Tumor cannot be evaluated | Tumor cannot be evaluated |
|  | Tis | NA | High-grade dysplasia, defined as malignant cells confined to the epithelium by the basement membrane |
|  | T0 | No signs of tumor | No evidence of primary tumor |
|  | T1 | NA | Lamina propria, muscularis mucosae, or submucosa |
|  | T2 | NA | Muscularis propria |
|  | T3 | Epithelium/submucosa (mucosal disease) | Adventitia |
|  | T4a | Deep soft tissue, cartilage, bone or overlying skin | Pleura, pericardium, azygos vein, diaphragm, or peritoneum |
|  | T4b | Brain, dura, skull base, lower cranial nerves, masticator space, carotid artery, prevertebral space, mediastinal structures, cartilage, skeletal muscle or bone | other adjacent structures, such as the aorta, vertebral body, or airway |
| N | Nx | Regional lymph nodes cannot be evaluated | Regional lymph nodes cannot be evaluated |
|  | N0 | No regional lymph nodes metastasis | No regional lymph nodes metastasis |
|  | N1 | Regional lymph node metastasis present | Metastasis in one or two regional lymph nodes |
|  | N2 | NA | Metastasis in three or six regional lymph nodes |
|  | N3 | NA | Metastasis in seven or more regional lymph nodes |
| M | M0 | No distant metastasis | No distant metastasis |
|  | M1 | Distant metastasis | Distant metastasis |
