# Supplementary material for: Clinicopathological Features, Staging Classification, and Clinical Outcomes of Esophageal Melanoma: Evaluation of a Pooled Case Series
Source: Front Oncol. 2022 Jul 1;12:858145. doi: 10.3389/fonc.2022.858145 (PMC9283823; doi:10.3389/fonc.2022.858145)
Supplement: Supplementary file 2 [file Table_2.docx]

Supplementary Table 2 Summary of study results

| Case number | Author | Age | Gender |
| --- | --- | --- | --- |
| 1 | A. Abdel Samie | 85 | F |
| 2 | Ahsan Malik | 70 | M |
| 3-7 | Amy A. Sanchez |  |  |
| 8 | Navarro-Ballester A. | 67 | M |
| 9 | Armin Gergera | 78 | M |
| 10-15 | Baozhong Li |  |  |
| 16 | Bernard Maroy | 65 | M |
| 17 | Butte JM | 51 | F |
| 18 |  | 59 | M |
| 19-24 | Daisuke Izumi |  |  |
| 25 | David A. | 75 | M |
| 26 | de Bruin GJ | 77 | F |
| 27 | Dennis KL | 47 | M |
| 28 | Frangi Caregnato A | 46 | F |
| 29 | Eric J Song | 67 | M |
| 30 | Eswar Tipirneni | 74 | M |
| 31 | Flávio Hiroshi Ananias Morita | 60 | M |
| 32 | Fujii K | 67 | F |
| 33-40 | H. Yu |  |  |
| 41 | Haruhisa Suzuki | 62 | M |
| 42 |  | 67 | M |
| 43 | Hiroyuki Miyatani | 64 | F |
| 44 | Houissa F | 78 | F |
| 45 | Hui Liu | 79 | M |
| 46 | I-Chen Wu, | 72 | F |
| 47 | Jarmila Prosvicovaa, | 55 | M |
| 48 | Jennifer C. Sinclair | 44 | F |
| 49 | Jennifer Rose Chapman Fredricks | 60 | M |
| 50-68 | Ji Yong Ahn |  |  |
| 69-74 | Jinfeng Zheng |  |  |
| 75 | Joana Machado | 87 | M |
| 76 | Joseph Sabat | 38 | M |
| 77 | Justin Kelly | 48 | M |
| 78-80 | JUSTYNA SZUMI |  |  |
| 81-90 | K. Harada |  |  |
| 91 | Kanamori N | 70 | M |
| 92 | Kazuhiro Imamura | 56 | F |
| 93 | Kenro Kawada | 64 | F |
| 94 | Khek Yu Ho | 48 | F |
| 95 | Sang Gyun Kim | 61 | F |
| 96 | Kyoko Inadomi | 57 | M |
| 97 | Kyoko Inadomi | 73 | M |
| 98-107 | Rupert Langer |  |  |
| 108 | Laura Granel-Villach | 67 | M |
| 109 | Lawrence J. Brandt | 72 | M |
| 110 | Lawrence J. Brandt | 70 | M |
| 111 | Lin Wang | 65 | M |
| 112 | Luoluo Yang | 63 | F |
| 113 | M. C. C. M. Hulshof | 77 | M |
| 114 |  | 83 | F |
| 115 | Massimo Raimondo | 74 | M |
| 116 | MEI WANG | 71 | F |
| 117 | Michele Bisceglia | 69 | M |
| 118 | Ming-Liang Lu | 48 | M |
| 119 | Min-Jung Kang | 56 | M |
| 120 | Mohammad Jaragh | 70 | F |
| 121 | Narendra Singh Choudhary | 50 | M |
| 122 | nicola solari | 75 | F |
| 123 | Nirmalkumar A. | 69 | M |
| 124 | Noriaki Kanamori | 70 | M |
| 125 | Oya Yonal | 89 | F |
| 126 | SATOSHI MAMORI | 62 | M |
| 127 | Pei-Ru Wu | 72 | M |
| 128 | Qingxi Yu, | 61 | M |
| 129 | Quor Meng Leong | 64 | F |
| 130 | Robin Rivera Irigoin | 47 | M |
| 131 |  | 65 | M |
| 132 | Sahar A. Saddoughi | | |
| 133 |  |  |  |
| 134 | Shaohua Wang | 48 | M |
| 135-146 | Shaohua Wang |  |  |
| 147 | Shi Wang | 82 | F |
| 148-158 | Shugeng Gao(total 17 cases, 6 cases overlapped and ommited) |  |  |
| 159 | Slim Charfi | 54 | M |
| 160 | Soichiro YAMAMOTO | 75 | M |
| 161 | Tadashi Terada | 87 | F |
| 162 |  | 56 | M |
| 163 | Spain | 69 | M |
| 164 | Takashi Oshiro1, | 73 | M |
| 165 | Takeshi Nonoshita | 71 | M |
| 166 | THOMAS FRIELING | 79 | M |
| 167 | Tzung-Ju L | 40 | F |
| 168 | Ueda Y | 68 | M |
| 169 |  | 57 | M |
| 170 | Vikas Gupta | 55 | M |
| 171 | GabrielWallis | 75 | M |
| 172 | Xueying Yang | 67 | M |
| 173 | Yang L | 50 | M |
| 174 | Yu-Bin Zhou | 71 | M |
| 175 | Yue-Ming Hu | 56 | F |
| 176 | Yirong Sim | 59 | M |
| 177 | Yun-Hong Li | 65 | F |
| 178 | zhang zhiyang | 64 | F |
| 179 |  | 58 | F |
| 180 | Daisuke Izumi | 74 | M |
| 181 | Shida A | 65 | F |
| 182 | Isozaki T | 46 | M |
| 183 | Kato T | 71 | M |
| 184 | Cardeña R | |  |
| 185 | Motoyama S | 53 | M |
| 186-206 | Sun HY |  |  |
| 207 | LEE | 61 | M |
| 208 | O'Sullivan J | 57 | M |
| 209 | Hiroyuki Ohnuma | 70 | M |
| 210 |  | 71 | M |
| 211 |  | 80 | M |
| 212 | Shinichiro Imai | 74 | M |
| 213 | Tian Zhao | 63 | M |
| 214 | Hyun Ho Choi | 65 | M |
| 215 | Sho Fukuda | 66 | M |
| 216 | Shota Kuwabara | 70 | F |
| 217 | Hani Katerji | 82 | F |
| 218 | Jingjing Li | 59 | F |
| 219 | Yutaka Hirayama | 77 | F |
| 220 | KEN SASAKI | 60 | M |
| 221 | JUNYA KOBAYASHI | 68 | M |
| 222 | Pauline Rochefort | 75 | M |
| 223 | Chunxiang Ling | 62 | M |
| 224 | Ruo-Xi Zhang | 49 | F |
| 225-244 | Jerzy Lasota |  |  |
| 245 | Naomichi Koga | 86 | F |
| 246-251 | Masayuki Urabe |  |  |
| 252 | Ji Li | 64 | M |
| 253 | Koki Nakamura | 67 | M |
| 254-273 | Hengchi Chen |  |  |
| 274 | Yasmine Hussein Agha | 90 | M |
| 275 | Kenichi Iwasaki | 74 | M |
| 276 | Fumitaka Endo | 70 | M |
| 277 | Ryoichi Tsukamoto | 74 | M |
| 278 | Yang Tang | 60 | F |
| 279 | Yu-Ming Chu | 78 | M |
| 280 | Shingo Ito | 80 | F |
| 281-286 | TADAYOSHI HASHIMOTO |  |  |
| 287 | Yuki HATTORI | 40 | M |
